# Assembly of the Mitochondrial Genome in the Campanulaceae Family Using Illumina Low-Coverage Sequencing

**DOI:** 10.3390/genes9080383

**Published:** 2018-07-30

**Authors:** Hyun-Oh Lee, Ji-Weon Choi, Jeong-Ho Baek, Jae-Hyeon Oh, Sang-Choon Lee, Chang-Kug Kim

**Affiliations:** 1 Phyzen Genomics Institute, Seongnam 13558, Korea; dlgusdh88@phyzen.com (H.-O.L.); sclee0923@phyzen.com (S.-C.L.); 2 Department of Plant Science, Seoul National University, Seoul 08826, Korea; 3 Postharvest Technology Division, National Institute of Horticultural and Herbal Science, Wanju 55365, Korea; jwcnpri@rda.go.kr; 4 Gene Engineering Division, National Institute of Agricultural Sciences, RDA, Jeonju 54874, Korea; firstleon@korea.kr; 5 Genomics Division, National Institute of Agricultural Sciences, RDA, Jeonju 54874, Korea; jhoh8288@korea.kr

**Keywords:** Campanulaceae, *Platycodon grandiflorus*, *Codonopsis lanceolata*, mitochondrial genome

## Abstract

*Platycodon grandiflorus* (balloon flower) and *Codonopsis lanceolata* (bonnet bellflower) are important herbs used in Asian traditional medicine, and both belong to the botanical family Campanulaceae. In this study, we designed and implemented a de novo DNA sequencing and assembly strategy to map the complete mitochondrial genomes of the first two members of the Campanulaceae using low-coverage Illumina DNA sequencing data. We produced a total of 28.9 Gb of paired-end sequencing data from the genomic DNA of *P. grandiflorus* (20.9 Gb) and *C. lanceolata* (8.0 Gb). The assembled mitochondrial genome of *P. grandiflorus* was found to consist of two circular chromosomes; the master circle contains 56 genes, and the minor circle contains 42 genes. The *C. lanceolata* mitochondrial genome consists of a single circle harboring 54 genes. Using a comparative genome structure and a pattern of repeated sequences, we show that the *P. grandiflorus* minor circle resulted from a recombination event involving the direct repeats of the master circle. Our dataset will be useful for comparative genomics and for evolutionary studies, and will facilitate further biological and phylogenetic characterization of species in the Campanulaceae.

## 1. Introduction

The large angiosperm Campanulaceae family comprises five subfamilies and over 2300 species. Members of this family—predominantly herbaceous perennials with capsular fruits—are found in almost all habitats [1]. Several Campanulaceae species have been used as medicinal plants in Asia. *Platycodon grandiflorus* (balloon flower) has distinct pharmacological effects in the treatment of coughs, excessive phlegm, and sore throats. Its root, called doraji in Korea, jiegeng in China, and kikyo in Japan, has been widely used as a traditional medicine in these countries [2]. In addition, *P. grandiflorus*, which is also used as a vegetable and an ornamental plant, shows strong ecological adaptability facilitated by resistance to drought, cold, and disease [3,4]. *Codonopsis lanceolata* (bonnet bellflower) exhibits various health benefits including antioxidant, antimicrobial, anti-inflammatory, and immune-modulating properties. The root of *C. lanceolata* has been used in traditional medicine to treat various conditions and symptoms, including bronchitis, coughs, and lung injury [5].

In *P. grandiflorus* and *C. lanceolata*, most studies have reported on their pharmacological effects and chloroplast genome [2,4]. However, the mitochondrial genome has not been reported for the Campanulaceae family including *P. grandiflorus* and *C. lanceolata*. The plant mitochondrial genome is an essential organelle that ranges from 200 to 700 kb in size, with some exceeding 1 Mb [6], and typically contain abundant repeated sequences [7]. These dynamic structures are useful for molecular ecology, evolutionary biology, and phylogenetic studies. Recently, next-generation sequencing (NGS) and related bioinformatics tools have been providing improved strategies for assembling genomes in plant species [8,9]. However, the complexed mitochondrial genomes of plants are more difficult to analyze than animal species [10,11]. In this study, first in Campanulaceae family, we generated the complete mitochondrial genomes of *P. grandiflorus* and *C. lanceolata* using a de novo NGS strategy without reference sequence and characterized the features of the genomes.

## 2. Materials and Methods

### 2.1. Plant Materials and Whole-Genome Sequencing

*Platycodon grandiflorus* (voucher # IT105900) and *C. lanceolata* (voucher # IT239919) were obtained from the National Institute of Horticultural and Herbal Science (NIHHS, Wanju, Korea). Total genomic DNAs were extracted from fresh leaves using a modified cetyltrimethylammonium bromide (CTAB) method [12]. DNA quality and concentration were examined using a spectrophotometer and a 2100 Expert Bioanalyzer (Agilent Technologies, Santa Clara, CA, USA). Paired-end (PE) libraries with an insert size of 270 to 700 bp were constructed according to the standard Illumina PE protocol and sequenced using an Illumina MiSeq platform (Illumina, San Diego, CA, USA) by Macrogen Biotechnology Center (Macrogen Inc., Seoul, Korea).

### 2.2. Assembly of Mitochondrial Genomes

**Figure 1:**
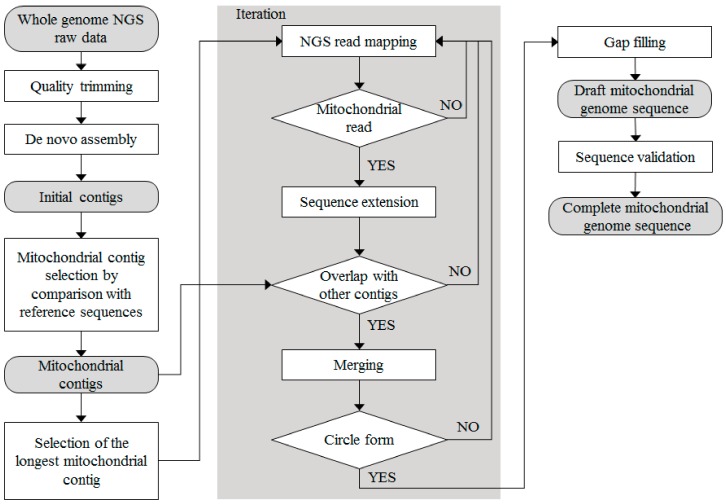
Pipeline for de novo assembly of mitochondrial genomes in the Campanulaceae family using Illumina, low-coverage, whole-genome next-generation sequencing (NGS) data.

In the assembling for organelle genome, high-quality read sequences can be obtained using low coverage data (0.5 to 5× based on the nuclear genome) [13]. In the schematic pipeline for de novo assembling (Figure 1), first, low-coverage PE data were extracted (Supplementary Table S1).

Second, PE data were filtered to obtain high-quality read sequences with Phred scores > 20 using the quality trim tool in the CLC Assembly Cell package ver. 4.2 (CLC Inc., Aarhus, Denmark). Third, four de novo assemblers were tested: Celera ver. 8.2 [14], SOAPdenovo ver. 2.02 [15], and SPAdes ver. 3.8 [16], CLC assembly Cell package (Supplementary Table S2). Fourth, high-quality read sequences were de novo assembled using the CLC assembly Cell package with the following parameters: distance between forward start and reverse end reads ranging from 300 to 700 bp; similarity fraction = 0.8; length fraction = 0.5. Fifth, mitochondrial contigs were selected using the mitochondrial genome sequence of *Ipomoea nil* [17] and the NUCmer tool [18]. Sixth, the contigs were extended by read mapping using the CLC reference mapper tool (CLC Inc, Denmark) with the same parameters as used for the de novo assembly. Seventh, mismatched assembly events were removed using BLASTN searches against the National Center for Biotechnology Information (NCBI) non-redundant nucleotide database. Finally, our processes generated a circular mitochondrial genome sequences whose gaps were completely filled using nucleotide sequences with the highest depth after read mapping.

### 2.3. Validation of Mitochondrial Genomes

The draft mitochondrial genome sequences were validated by PE read mapping and PCR amplification. For validation using PE read mapping, PE reads used for assembly were mapped on the mitochondrial genome sequences. Depth of mapped reads was investigated using the CLC Assembly Cell package and visualized using Microsoft Excel. Consistency and connectivity of mapped reads were confirmed. In addition, junctions between seed contigs were confirmed. For validation using PCR amplification, 12 primers were designed in the *P. grandiflorus* (Supplementary Table S3). The six primers were designed within contig sequences of >1 kb, and another six primers were designed at junction regions between contigs or within contig sequences of <1 kb. All PCRs were performed using the DNA Engine Tetrad 2 Peltier Thermal Cycler (BIO-RAD, Hercules, CA, USA). The following PCR conditions were used: 95 °C for 5 min; 35 cycles of 95 °C for 30 sec, 58 °C for 30 sec, and 72 °C for 1 min; and 72 °C for 10 min.

### 2.4. Annotation of Mitochondrial Genome

Mitochondrial genomes were annotated using the GeSeq program (Max Planck Institute, Golm, Germany). Three species were used as references for gene prediction: *Helianthus annuus* (KF815390) [19], *Daucus carota* (JQ248574) [8], and *Vaccinium macrocarpon* (KF386162) [20]. Ambiguous gene positions were manually corrected using NCBI BLASTN searches and the Artemis annotation tool (http://www.sanger.ac.uk/science/tools/artemis). Circular maps of mitochondrial genomes with the annotation information were drawn using the OGDRAW program [21]. The complete mitochondrial genome sequences assembled in this study were deposited into the GenBank database under KX887331, MG775429, and MG775430.

### 2.5. Analysis of Repetitive Sequences

Repetitive sequences in the mitochondrial genomes were searched using the REPuter program [22], with a minimum repeat size of 20 bp and four selected repeat types (forward, reverse, complement, and reverse complement). The detected repetitive sequences were divided into two groups based on a size cutoff of 100 bp. Repetitive sequences of >100 bp were depicted on the circular genome maps using the ClicO FS (Codon Genomics Inc., Selangor, Malaysia) program. Tandem repeat sequences were detected using the Tandem Repeats Finder software (BU-Bioinfomatics, MA, USA) with the following parameters: alignment parameters of 2 bp match, 2 bp mismatch, and 7-bp insertion and deletions (InDels); minimum alignment score of 50; maximum period size of 500; and maximum tandem repeat array size of 2 bp.

### 2.6. Bayesian Phylogenetic Analysis

Phylogenetic analysis based on Bayesian inference was performed using 14 protein-coding sequences (*atp1*, *ccmB*, *ccmC*, *ccmFc*, *ccmFn*, *cox1*, *cox3*, *matR*, *nad3*, *nad6*, *nad7*, *rps4*, *rps12*, and *rps13*) conserved in the mitochondrial genomes of 16 species in the asterids group including *Ajuga reptans* (KF709392), *Asclepias syriaca* (KF541337), *Boea hygrometrica* (JN107812), *Capsicum annuum* cultivar Jeju (KJ865410), *Castilleja paramensis* (KT959112), *C. lanceolata* (MG775430), *D. carota* (JQ2485740), *H. annuus* (KF815390), *Hyoscyamus niger* (KM207685), *I. nil* (AP017303), *Mimulus guttatus* (JN098455), *Nicotiana tabacum* (BA000042), *P. grandiflorus* (KX887331), *Rhazya stricta* (KJ485850), *Salvia miltiorrhiza* (KF177345), and *V. macrocarpon* (KF386162). In order to obtain a phylogenetic tree, BEAST ver. 2.4 program was employed and Markov chain Monte Carlo runs were performed for 1,000,000 chain length and sampling frequency set to every 10,000 steps [23]. The phylogenetic tree was annotated as a maximum clade credibility tree using the TreeAnnotator ver. 2.4 [23]. 

## 3. Results

### 3.1. Pipeline for the Assembly

We generated the complete mitochondrial genomes of *P. grandiflorus* and *C. lanceolata*, via de novo assembly using NGS data without reference genomes. The raw PE data were produced from *P. grandiflorus* (20.9 Gb, 79.1× coverage) and *C. lanceolata* (8.0 Gb, 161.4× coverage), respectively. The high quality 23,971,262 (*P. grandiflorus*, 95.9% of all sequences) reads and 23,424,520 (*C. lanceolata*, 87.7% of all sequences) reads were obtained after quality trimming (Supplementary Table S1). When four de novo assemblers were compared with two classified contigs group, SOAPdenovo assembler generated the smallest number of 58 contigs and SPAdes assembler resulted in the longest 1.8 Mb contig length in the *P. grandiflorus*. However, N50 and L50 statistics show that SOAPdenovo and CLC assembler have better performance than other assemblers. Therefore, we performed the assembly using the CLC assembler (Supplementary Table S2).

### 3.2. Complete Mitochondrial Genomes

**Figure 2:**
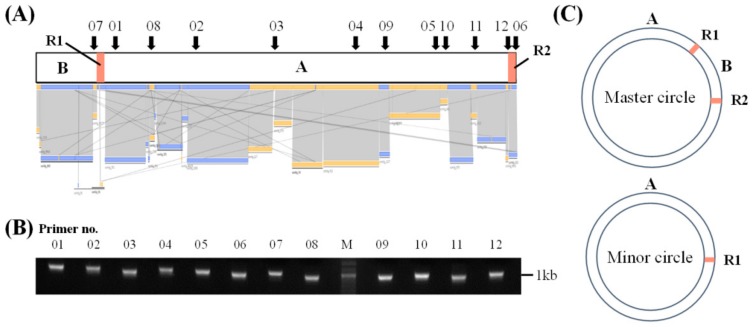
Assembly of the mitochondrial genome sequences of *Platycodon grandiflorus*. (**A**) Mitochondrial contigs that were utilized in the assembly of the complete mitochondrial genome. Blue and yellow bars indicate mitochondrial contigs used for extension, gap-filling, and merging. Black arrows indicate target locations of PCR primers used for validation. Primers 01 through 06 annealed within >1 kb contigs, and primers 07 through 12 annealed at junction regions between contigs or within <1 kb contigs; (**B**) PCR validation of the assembled master circle mitochondrial sequence. The 12 primer sets were used for genomic DNA PCR analysis. The sequences of PCR products of expected size were confirmed to be the same as the assembled sequences. M indicates the DNA ladder; (**C**) Schematic diagrams of the master circle and the minor circle of the *P. grandiflorus* mitochondrial genome, showing mitochondrial heteroplasmy in *P. grandiflorus*. The master circle consists of two regions, A and B, separated by the 21.8 kb large repeats R1 and R2. The minor circle contains only region A.

**Figure 3:**
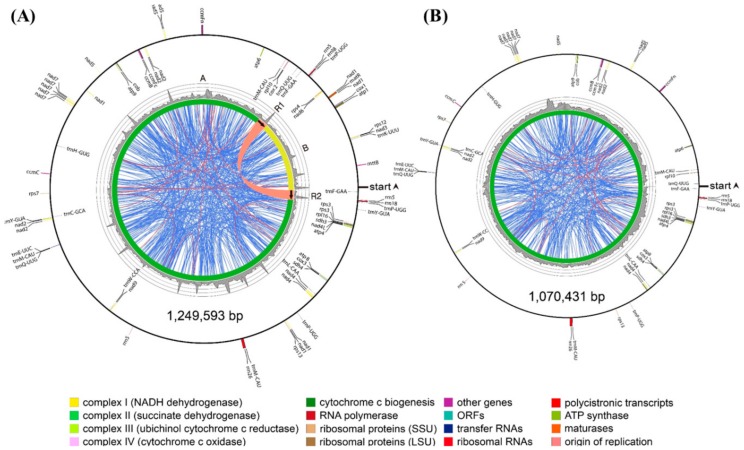
Maps of the *P. grandiflorus* mitochondrial genome. (**A**) Master circle; (**B**) Minor circle. Direction of gene transcription is represented by the location of the genes on the inside (clockwise) or the outside (counterclockwise) of the outermost circle. Inner histogram indicates average coverage depth of reads mapping to the mitochondrial genome sequence (each grey circle represents 50×, with a maximum of 200×). Innermost circle represents the structural scheme. Repeats of >100 bp are indicated by connecting orange lines and repeats of <100 bp are indicated by connecting blue lines. The thick orange line connects the largest 21.8 kb R1 and R2 repeat regions in the master circle. Tandem repeats are not shown.

In the *P. grandiflorus*, mitochondrial genome consists of two circular chromosomes, master circle of 1,249,593 bp and minor circle of 1,070,431 bp (Supplementary Table S4). The master circle harbored a pair of large identical 21.8 kb repeats (R1 and R2), which divided the genome into two regions (Figure 2A,C and Figure 3A). The minor circle contained region A and a single copy of the large repeat, both of which were identical to the ones found in the master circle (Figure 2C and Figure 3B).

**Figure 4:**
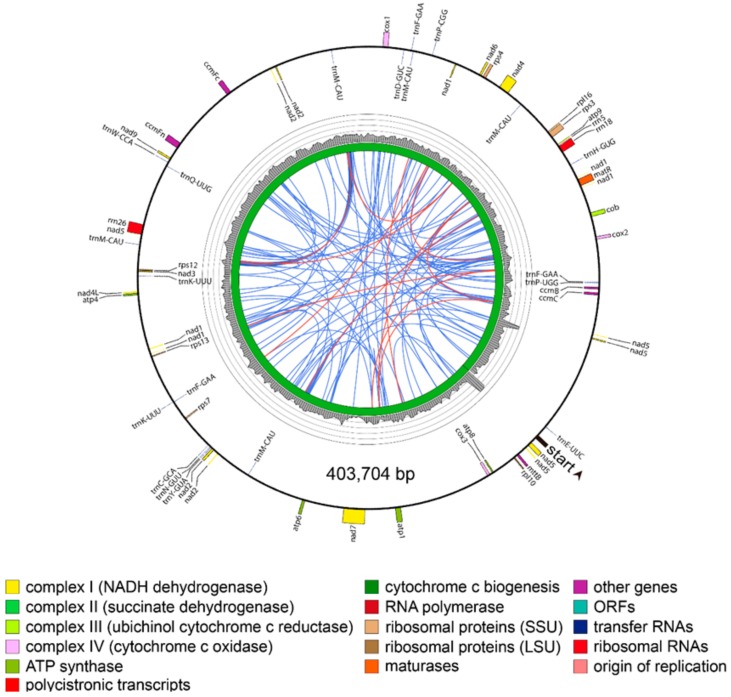
Mitochondrial genome map of *Codonopsis, lanceolata*. Direction of transcription is represented by the location of the respective genes on the inside (clockwise) or the outside (counterclockwise) of the outermost circle. Inner histogram indicates average coverage depth of reads mapped to the mitochondrial genome sequence (each grey circle represents 60×, with a maximum of 300×). Innermost circle indicates the structural scheme. Repeats of >100 bp are indicated by connecting orange lines and repeats of <100 bp are indicated by connecting blue lines. Tandem repeats are not shown.

Thus, due to the presence of region B and the R2 repeat in the master circle, the two *P. grandiflorus* mitochondrial genome structures shared 85.7% sequence similarity (Supplementary Figure S1). In the *C. lanceolata*, mitochondrial genome was revealed as a 403,704 bp circle assembled using 11 mitochondrial contigs, with no large repeats detected (Figure 4).

In order to validate the draft mitochondrial genome, first, sequence of the *P. grandiflorus* master circle was validated by PCR amplification and nucleotide sequencing using 12 primer pairs designed to anneal within junction regions and contigs (Figure 2B). Finally, the sequences of the two circles were validated by NGS read mapping, demonstrating consistency and connectivity of mapped reads at the whole-genome level and junctions between contigs (Supplementary Figure S1). In the *C. lanceolata*, mitochondrial genome was validated by consistency and connectivity of the mapped reads (Supplementary Figures S2 and S3). Although the *P. grandiflorus* and *C. lanceolata* shared <21.9% overall sequence similarity, the coding regions showed substantially higher similarity relative to the intergenic regions. These multi-validation steps confirm the reliability of our assembly pipeline, as well as the reliability of the complete mitochondrial genome sequence.

### 3.3. Annotated Genes in the Mitochondrial Genomes

In the *P. grandiflorus*, we identified 56 and 42 putative genes within the master and minor circles of the mitochondrial genome, respectively. In addition, the master and minor circles contained 46 and 36 non-redundant genes, respectively. In the *C. lanceolata*, mitochondrial genome contained a total of 54 genes, of which 47 were identified as non-redundant (Supplementary Table S4). Total of 23 redundant genes were detected by only dispersed repeats in *P. grandiflorus* and *C. lanceolata*. These redundant genes were found in the master circle of *P. grandiflorus* (*rrn18*, *rrn5_2 copy*, *trnF-GAA*, *trnM-CAU_2 copy*, *trnP-UGG_2 copy*, *trnQ-UUG*, *trnW-CCA*), minor circle of *P. grandiflorus* (*rrn5*, *trnM-CAU_2 copy*, *trnP-UGG*, *trnQ-UUG*, *trnY-GUA*), and *C. lanceolata* (*trnF-GAA_2 copy*, *trnK-UUU*, *trnM-CAU_4* copy), respectively.

In the *P. grandiflorus* master circle, total 46 non-redundant genes accounted for 3.23% of the sequence, comprised 32 protein-coding genes, 11 transfer RNAs (tRNAs), and 3 ribosomal RNAs (rRNAs). However, the minor circle lacked 10 genes (i.e., *nad1*, *nad3*, *nad6*, *cox1*, *atp1*, *rps4*, *rps12*, *matR*, *mttB*, and *trnK-UUU*) compared to the master circle. In the *C. lanceolata*, non-redundant genes accounted for 9.91% of the sequence, comprising 31 protein-coding genes, 13 tRNAs, and three rRNAs (Table 1). In addition, we compared the reported mitochondrial genome of *H. annuus* (sunflower), which is a closely related species belonging to the asterids group [19].

The 27 common genes are conserved in the three species (i.e., *P. grandiflorus*, *C. lanceolata*, and *H. annuus*). Most of the genes were associated with the electron transport chain, cytochrome c biogenesis, and protein synthesis. The unique genes (i.e., *sdh3* and *sdh4*) of *P. grandiflorus* were found in the encoding subunits of the succinate dehydrogenase complex (Complex II) category. The trans-spliced protein-coding genes were found in the *P. grandiflorus* master circle (*nad1* and *nad2*), minor circle (*nad2*), and *C. lanceolata* (*nad1* and *nad2*). The *mttB* gene, which catalyzes the transfer of a methyl group from trimethylamine, was found in the master circle of *P. grandiflorus* and *C. lanceolate* (Table 1).

### 3.4. Repetitive Sequences in the Mitochondrial Genomes

Repetitive sequences consist mostly of dispersed repeats and tandem repeats, and they can vary in size in plant mitochondrial genomes [7]. The dispersed repeats are segments of DNA that occur multiple times at more or less random positions in the genome, and tandem repeats are small segments of DNA repeated one after another [24,25]. The dispersed repeats and tandem repeats were investigated in terms of presence and frequency in the *P. grandiflorus* and *C. lanceolata*.

The *P. grandiflorus* master circle contained 635 dispersed repeats covering 7.4% (92,902 bp) of the genome, and these repeats mostly ranged from 20 to 40 bp in size. Among the 19 repeats exceeding 100 bp, the largest 21,751 bp repeats divided the master circle into two regions (Figure 3A). In contrast, the minor circle contained less dispersed repeats than the master circle. The 455 dispersed repetitive sequences of the minor circle covered 2.9% (31,448 bp) of its sequence, with 14 exceeding 100 bp in size. In the *C. lanceolata*, 121 dispersed repeats were found in relation to *P. grandiflorus*, covering 4.2% (16,944 bp) of the genome, which were reduced in both total number and length. In the *H. annuus*, distribution of dispersed repeat was similar to *P. grandiflorus* and *C. lanceolata* despite difference of total number and amount (Supplementary Table S5, Figure S4).

The tandem repeats were less common and were not detected at genome-specific frequencies, with similar levels observed in the mitochondrial genomes of all three species. These repeats mostly ranged from 10 to 30 bp in size and covered no more than 1500 bp of the analyzed genomes. Especially, most tandem repeats were present within intergenic regions (Supplementary Table S6, Figure S4).

### 3.5. Phylogenetic Relationships Based on Mitochondrial Sequences

**Figure 5:**
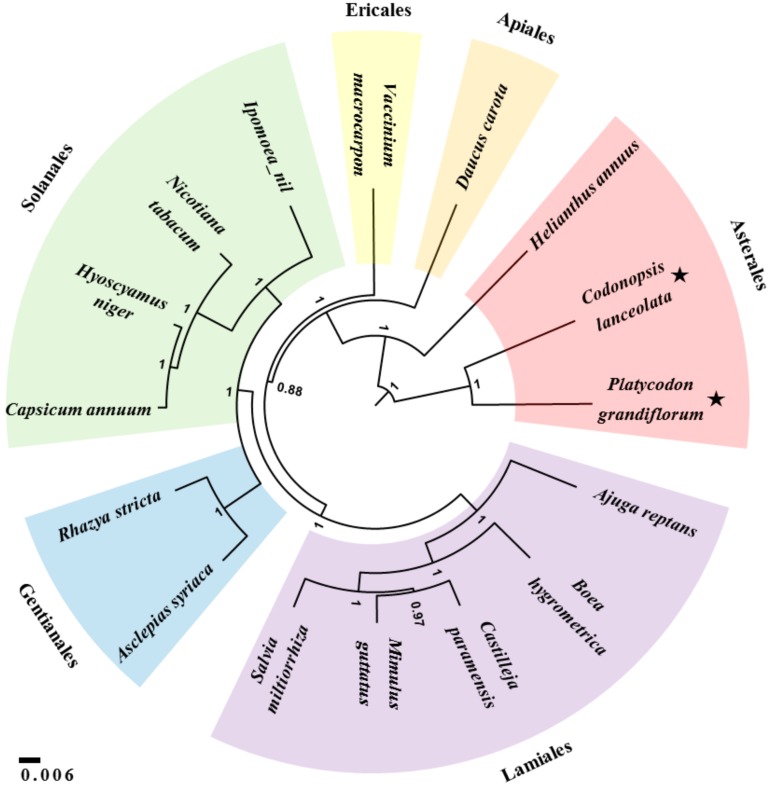
Phylogenetic relationship of 16 asterids determined using 14 conserved mitochondrial protein-coding sequences. Posterior probability distributions calculated by the BLAST program are indicated at the branch points. Scale bar represents the number of nucleotide substitution per site. The following mitochondrial genome sequences were used for the phylogenetic analysis: *A. reptans*, *A. syriaca*, *B. hygrometrica*, *C. annuum*, *C. paramensis*, *C. lanceolata*, *D. carota* subsp. sativus, *H. annuus*, *H. niger*, *I. nil*, *M. guttatus*, *N. tabacum*, *P. grandiflorus*, *R. stricta*, *S. miltiorrhiza*, and *V. macrocarpon*.

Phylogenetic analysis was performed using 14 conserved protein-coding sequences commonly found in mitochondrial genomes of 16 species belonging to the asterids group (Figure 5). The phylogenetic tree was divided into six groups that were consistent with the respective orders of the selected plants (i.e., Asterales, Apiales, Ericales, Gentianales, Lamiales, and Solanales).

## 4. Discussion

In this study, we first designed and implemented a de novo assembly pipeline to map the complete mitochondrial genomes of Campanulaceae family using NGS data. In order to find the optimal method of assembling, we compared the efficiency of four de novo assemblers. Ultimately, our method employed the CLC Assembly Cell assembler, which demonstrated superior performance with respect to N50 and L50 relative statistics. However, other plants have been generated using different assemblers, such as MITObim, Newbler, and PLATANUS [26,27,28,29], combination of assemblers [30]. Thus, we suggest that the assembly method is different depending on the NGS data type and complexity characteristics of each plant.

The *P. grandiflorus* and *C. lanceolata* mitochondrial genomes were within the 1.5 to 2000 kb range observed for reported mitochondrial genomes of 223 land plant species (Supplementary Figure S5). Although two species are in the same Campanulaceae family, mitochondrial genome sizes of *P. grandiflorus* were more than two times larger than the *C. lanceolata*. Substantial within-family differences have been observed between the members of other plant families. In the Cucurbitaceae family, cucumber and watermelon are 1556 kb and 379 kb, respectively [31,32]. Despite differences in overall size, the mitochondrial genomes of *P. grandiflorus* and *C. lanceolata* harbored similar numbers of coding genes, as reported in other plant mitochondrial genomes (Supplementary Figure S6). We revealed the presence of species-specific genes, as well as potential gene loss in *P. grandiflorus* and *C. lanceolata*. Interestingly, genes encoding subunits of the succinate dehydrogenase complex (Complex II), *sdh3* and *sdh4*, were found only in the master and minor circles of *P. grandiflorus*, whereas *mttB* gene was found only in the master circle of *P. grandiflorus* and in the *C. lanceolata* genome. These *sdh3* and *sdh4* genes were transferred from the mitochondrial genome to the nuclear genome during angiosperm evolution, and diverse angiosperm species have retained both genes in their mitochondrial genomes [33]. Therefore, *sdh3* and *sdh4* genes in *C. lanceolata* and *H. annuus* are likely present in the nuclear genomes of these two species. Overall, our findings regarding mitochondrial gene distribution underscored the dynamic nature of mitochondrial genomes.

Mitochondrial genomes of *P. grandiflorus* and *C. lanceolata* were characterized by high-repeat content and low gene density, properties that are typical of plant mitochondrial genomes. The differences of plant mitochondrial genomes can be shown by repetitive sequences and frequent recombination events [34]. The dispersed and tandem repeats were proportional to the size of the mitochondrial genome, and intergenic, region-specific repeats have likely contributed to increases in the complexity of mitochondrial genome [7,35]. The *H. annuus* has a similar distribution of repeats to mitochondrial genomes of *P. grandiflorus* and *C. lanceolata*, despite the differences in dispersed repeats. However, the localization of the dispersed repeats was similar in all mitochondrial genomes, with >90% of repeats associating with the intergenic regions in three species. Although there was a difference between the mitochondrial genome length and the dispersed repeats, three plant species (i.e., *P. grandiflorus*, *C. lanceolata,* and *H. annuus*) were similar in GC content, total gene number, and average gene length (Supplementary Table S4).

Interestingly, we identified a multipartite (i.e., master and minor circles) mitochondrial genome of *P. grandiflorus*. Multipartite mitochondrial genomes have been reported in some plants, animals, and fungi [36]. Generally, multipartite genome structure was generated using high-frequency recombination via repeated sequences, intramolecular homologous recombination, random genetic drift, and loss of mitochondrial single-strand binding protein [37,38]. In the heteroplasmy, in which divergent mitochondrial genotypes co-exist in a plant cell [39], *P. grandiflorus* is potentially present in additional types of mitochondrial genomes. However, in the multipartite mitochondrial genomes of *P. grandiflorus*, substantial differences exist in both sequence and structural levels with repeat sequences (Figure 2 and Figure 3). The comparison of sequence and linear genome map of *P. grandiflorus* indicates that the master and minor circle are almost identical, except for deletion of 179 kb, one direct repeat (R2 region), and related region (B region) in minor circle (Figure 2, Supplementary Figures S1 and S2). Therefore, we assume that the largest repeats of the master circle contributed to creation of the minor circle of *P. grandiflorus*. In additional validation, we are going to verify the heteroplasmy of *P. grandiflorus* using higher coverage and longer read sequencing data, under assembly conditions that can attenuate the issues associated with high-depth repeats.

Despite many phylogenetic analyses using chloroplast and nuclear sequences, such as *rbcL*, the internal transcribed spacer (ITS), and pentatricopeptide repeat (PPR) genes [40,41,42,43], the Campanulaceae family’s phylogenetic relationships have remained poorly characterized [44]. To date, mitochondrial sequences have not been utilized for phylogenetic analysis of the Campanulaceae family species. Although there are limited mitochondrial sequences, we sought to perform a mitochondrial, genome-based analysis using our newly assembled data. Unlike sequence and structural conversation of chloroplast genomes among plant species, mitochondrial genomes show much difference both at sequence and structural level due to abundance of repetitive sequences and frequent recombination (Figure 2, Figure 3 and Figure 4, Supplementary Figures S1–S3). Therefore, instead of whole mitochondrial genome sequence or total protein-coding sequences, we use the 14 conserved protein-coding sequences commonly present among 16 mitochondrial genomes of asterids group (Figure 5). The phylogenetic tree divided 16 species into six groups. The *P. grandiflorus* and *C. lanceolata* clustered the Asterales order group with *H. annuus*. The *D. carota* (carrot) demonstrated a sister relationship in the Apiales order. The phylogenetic relationship of *P. grandiflorus*, *C. lanceolata*, and other species was in agreement with established taxonomical classifications [40,45].

Overall, we first assembled the complete mitochondrial genomes in the Campanulaceae family; annotated genes will be useful for biology and plant evolution. In addition, our newly designed assembly pipeline will facilitate further analyses of plant mitochondrial genomes.

## Figures and Tables

**Table 1:** Annotated genes in the mitochondrial genomes of *P. grandiflorus* (including master and minor circle) and *C. lanceolata*, and comparison with *Helianthus annuus*.

Category ^1^	Common Genes	Unique Genes			
		*P. Grandiflorus*		*C. Lanceolata*	*H. Annuus*
		(Master)	Minor		
Complex I	*nad4L*, *nad9*	*nad1–4*, *nad6*, *nad7*	*nad2*, *nad4*, *nad7*	*nad1–7*	*nad3*, *nad5–6*
Complex II		*sdh3*, *sdh4*	*sdh3*, *sdh4*		
Complex III	*cob*				
Complex IV	*cox3*	*cox1*, *cox2*	*cox2*	*cox1*, *cox2*	*cox1*
Complex V	*atp4*, *atp6*, *atp8*, *atp9*	*atp1*		*atp1*	*atp1*
Cytochrome	*ccmB*, *ccmC*, *ccmFc*, *ccmFn*				
Large subunit	*rpl10*, *rpl16*				*rpl5*
Small subunit	*rps13*	*rps3*, *rps4*, *rps7*, *rps12*	*rps3*, *rps7*	*rps3*, *rps4*, *rps7*, *rps12*	*rps4*, *rps12*, *rps13*
Maturase		*matR*		*matR*	*matR*
Transport protein		*mttB*		*mttB*	
Ribosomal RNAs	*rrn5*, *rrn18*, *rrn26*				
Transfer RNAs	*trnC-GCA*, *trnE-UUC*, *trnF-GAA*, *trnH-GUG*, *trnM-CAU*, *trnP-UGG*, *trnQ-UUG*, *trnW-CCA*, *trnY-GUA*	*trnK-UUU*, *trnL-CAA*	*trnL-CAA*	*trnD-GUC*, *trnK-UUU*, *trnN-GUU*, *trnP-CGG*	*trnD-GUC*, *trnG-GCC*, *trnN-GUU*, *trnS-GCT*
Total genes	27	19	9	20	13

^1^ Complex I (NADH dehydrogenase), complex II (succinate dehydrogenase), complex III (ubiquinol cytochrome c reductase), complex IV (cytochrome c oxidase), complex V (ATP synthase), cytochrome c biogenesis, large subunit ribosomal proteins, and small subunit ribosomal proteins.

## Supplementary Materials

The following are available online at http://www.mdpi.com/2073-4425/9/8/383/s1, Figure S1: Sequence comparison between the master and the minor circles of the *P. grandiflorus* mitochondrial genome. Figure S2: Linear genome map and read depth of the mitochondrial genomes of *P. grandiflorus* and *C. lanceolata*. Figure S3: Mitochondrial genome sequence comparison between *P. grandiflorus* and *C. lanceolata*. Figure S4: Repetitive sequences in the mitochondrial genomes of *P. grandiflorus* (including master and minor circle), *C. lanceolata*, and *H. annuus*. Figure S5: Size distribution of mitochondrial genomes of land plant species, based on the NCBI Organelle Genome database (https://www.ncbi.nlm.nih.gov/genome/ browse/?report=5#!/organelles/). The 223 mitochondrial genomes of land plant species have been deposited into the database. Figure S6. Numbers of annotated genes in assembled mitochondrial genomes of land plants, as revealed by the NCBI Organelle Genome database. Table S1: Overview of raw and trimmed data. Table S2: Comparison of four assemblers with two contig group. Table S3: Primers used for assembly validation. Table S4: Features of assembled mitochondrial genomes of *P. grandiflorus* (including master and minor circle) and *C. lanceolata* with *H. annuus*. Table S5: Repeat regions identified in the *P. grandiflorus* (including master and minor circle), *C. lanceolata* and *H. annuus*. Table S6: Tandem repeat sequences identified in the *P. grandiflorus* (including master and minor circle), *C. lanceolata,* and *H. annuus*.
