# Supplementary material for: Assembly of the Mitochondrial Genome in the Campanulaceae Family Using Illumina Low-Coverage Sequencing
Source: Genes (Basel). 2018 Jul 30;9(8):383. doi: 10.3390/genes9080383 (PMC6116063; doi:10.3390/genes9080383)
Supplement: Supplementary file 1 [file genes-09-00383-s001.zip › Supplementary-figures-final.pdf]

genes

Supplementary Figures

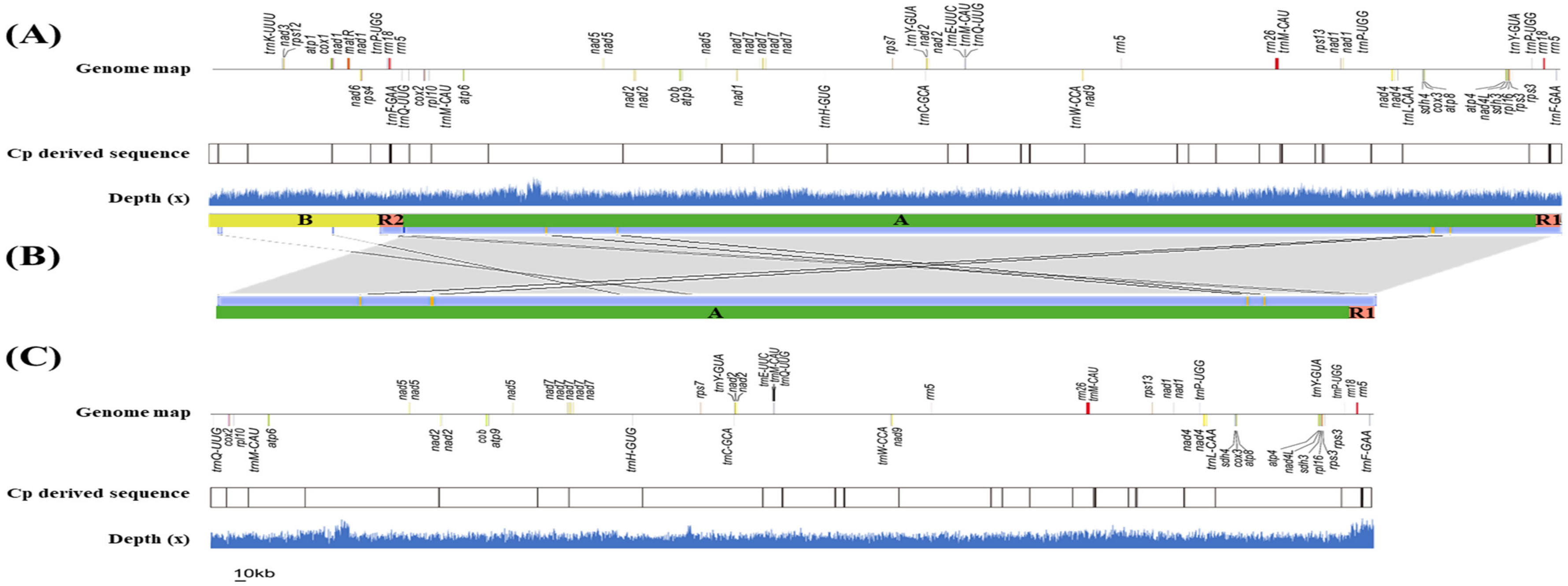

Supplementary Figure S1. Sequence comparison between the master and the minor circles of the *P. grandiflorus* mitochondrial genome. **(A)** The topmost line indicates the linear genome map of the master circle of *P. grandiflorus*. The genes above and below the line indicate genes present on the forward and reverse strands, respectively. The middle bar represents chloroplast (Cp)-derived sequences in the *P. grandiflorus* mitochondrial genome. Chloroplast genome-derived sequences were found by BLASTN searches against the chloroplast genome of *P. grandiflorus* (GenBank KX887331). Mitochondrial sequences >80% similar to the chloroplast sequences were considered to be derived from the chloroplast genome and are shown as black vertical lines. The bottom histogram indicates depth distribution of mapped reads. **(B)** Sequence homology between master and minor circles as determined using NUCmer program (the minimum length of a single match of 100 bp) in the MUMmer package. Homologous sequences are indicated by the grey lines connecting the two genome sequences. Genome structures of the two genomes were also indicated. **(C)** Genome map, chloroplast genome-derived sequences, and mapped read depth of the minor circle of the mitochondrial genome of *P. grandiflorus*.

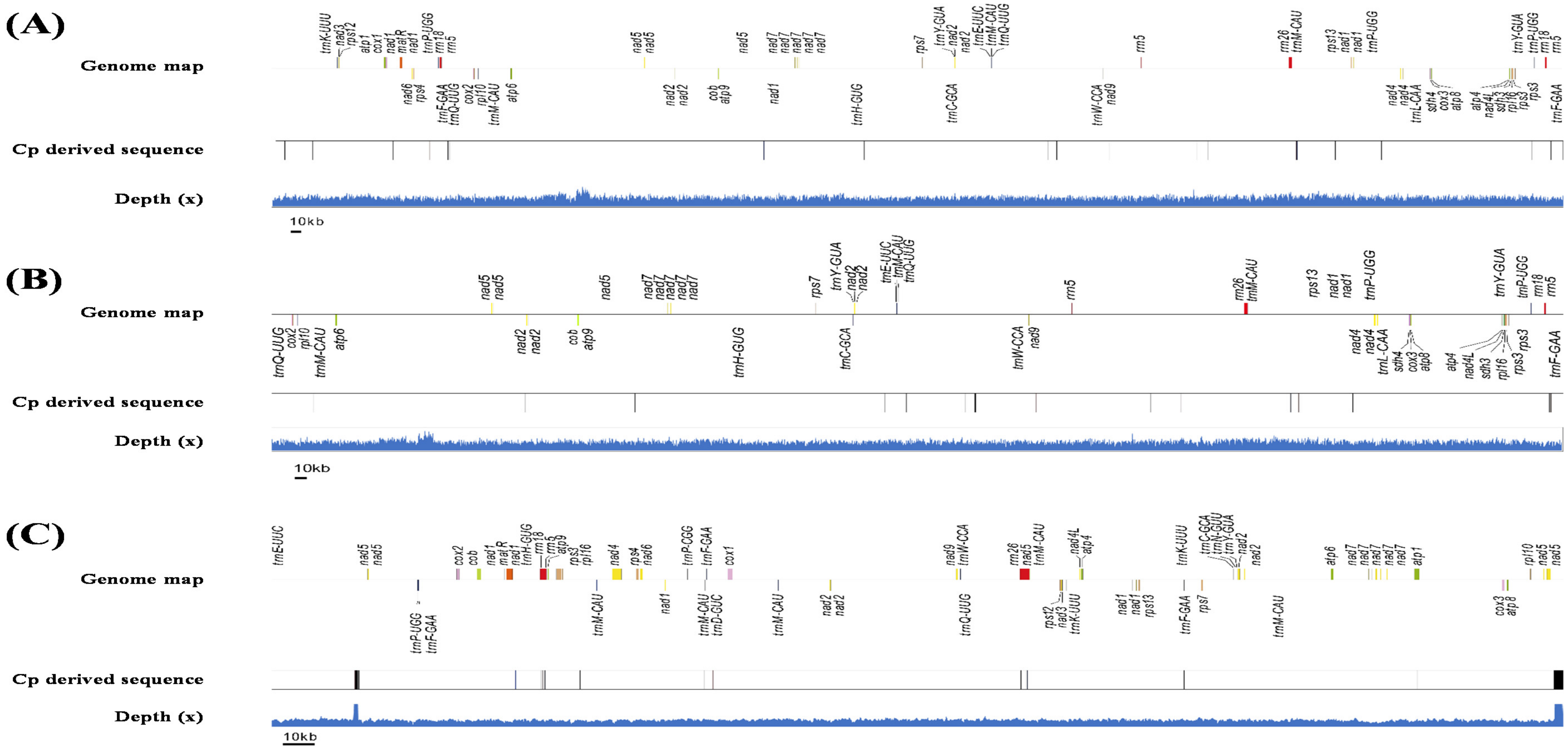

Supplementary Figure S2. Linear genome map and read depth of the mitochondrial genomes of *P. grandiflorus* and *C. lanceolata.* **(A)** The master circle of the *P. grandiflorus* mitochondrial genome. **(B)** The minor circle of the *P. grandiflorus* mitochondrial genome. **(C)** Mitochondrial genome of *C. lanceolata*. In each panel, the topmost line indicates the linear genome map. The genes above and below the line indicate genes present on the forward and reverse strands, respectively. The middle bar represents chloroplast (Cp)-derived sequences in the mitochondrial genome. Chloroplast genome-derived sequences were found by BLASTN searches against chloroplast genomes of *P. grandiflorus* (GenBank KX887331) and *C. minima* (GenBank KY587457). Mitochondrial sequences >80% similar to chloroplast sequences were considered to be derived from the chloroplast genome and are shown as black vertical lines. The bottom histogram indicates depth distribution of mapped reads.

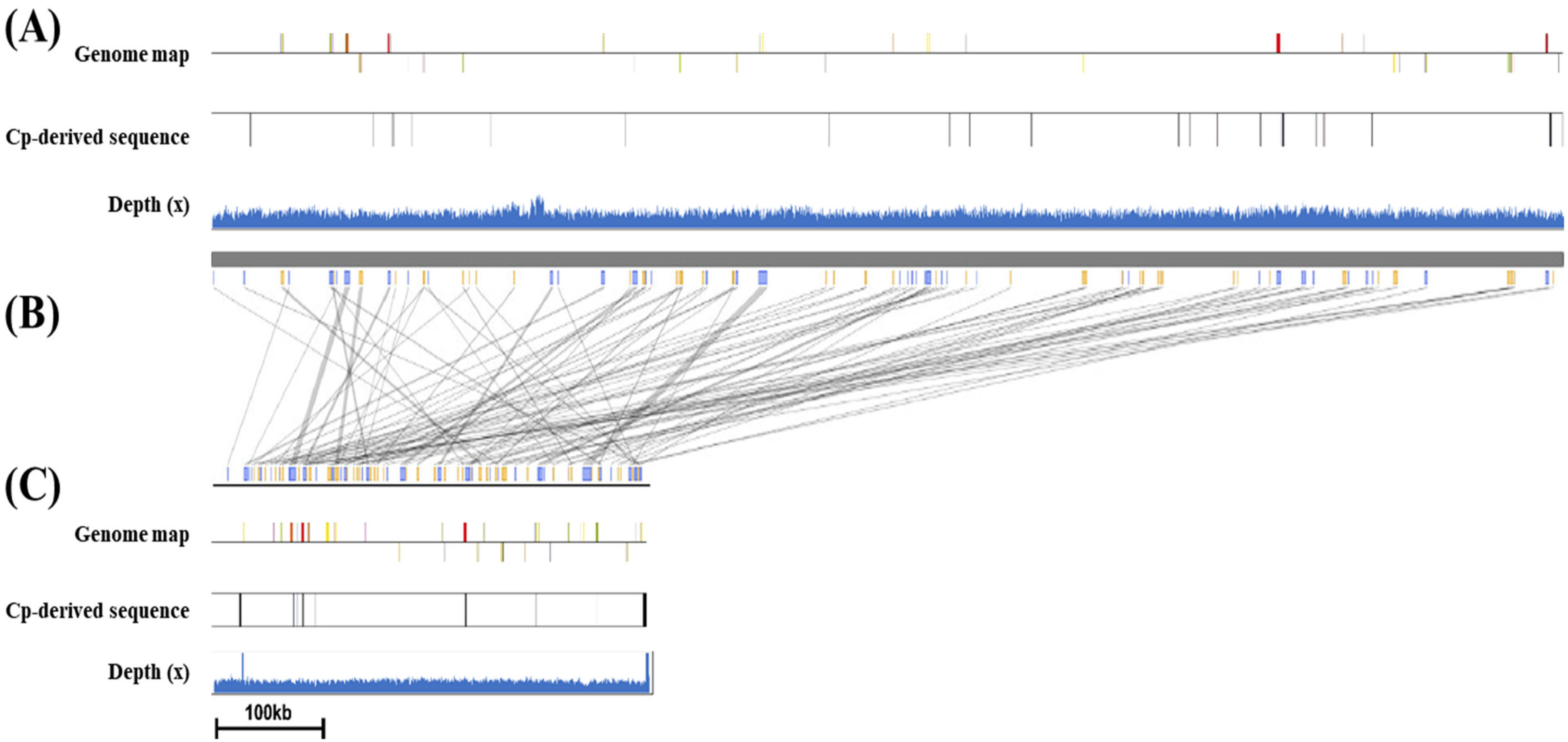

Supplementary Figure S3. Mitochondrial genome sequence comparison between *P. grandiflorus* and *C. lanceolata*. **(A)** The topmost line indicates the linear genome map of the master circle of the mitochondrial genome in *P. grandiflorus*. The genes above and below the line indicate genes present on the forward and reverse strands, respectively. The middle bar represents chloroplast (Cp)-derived sequences in the *P. grandiflorus* mitochondrial genome. The bottom histogram indicates depth distribution of mapped reads. **(B)** Sequence homology between mitochondrial genomes of *P. grandiflorus* and *C. lanceolata* using the BLASTZ program (MSP cutoff of 10,000 and E-value of 1E-06). Homologous sequences are indicated by the grey lines connecting the two genome sequences. **(C)** Genome map, chloroplast genome-derived sequences, and read depth of the *C. lanceolata*

mitochondrial genome.

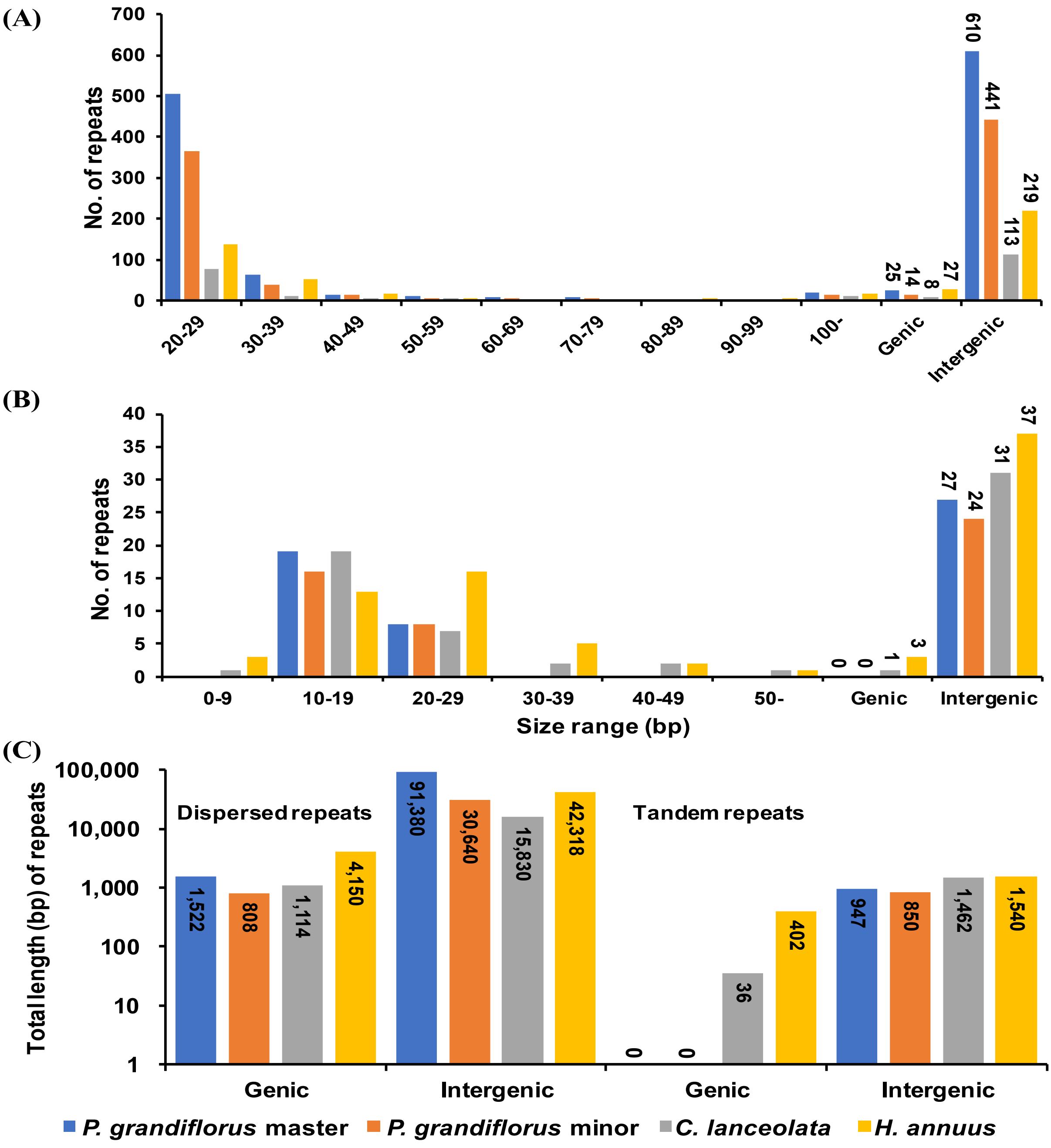

Supplementary Figure S4. Repetitive sequences in the mitochondrial genomes of *P. grandiflorus* (including master and minor circle), *C. lanceolata*, and *H. annuus*. **(A)** Number and size distribution of dispersed repeats identified by REPuter program. **(B)** Number and size distribution of tandem repeats identified by Tandem repeat finder program. **(C)** Total length (bp) of dispersed or tandem repeats.

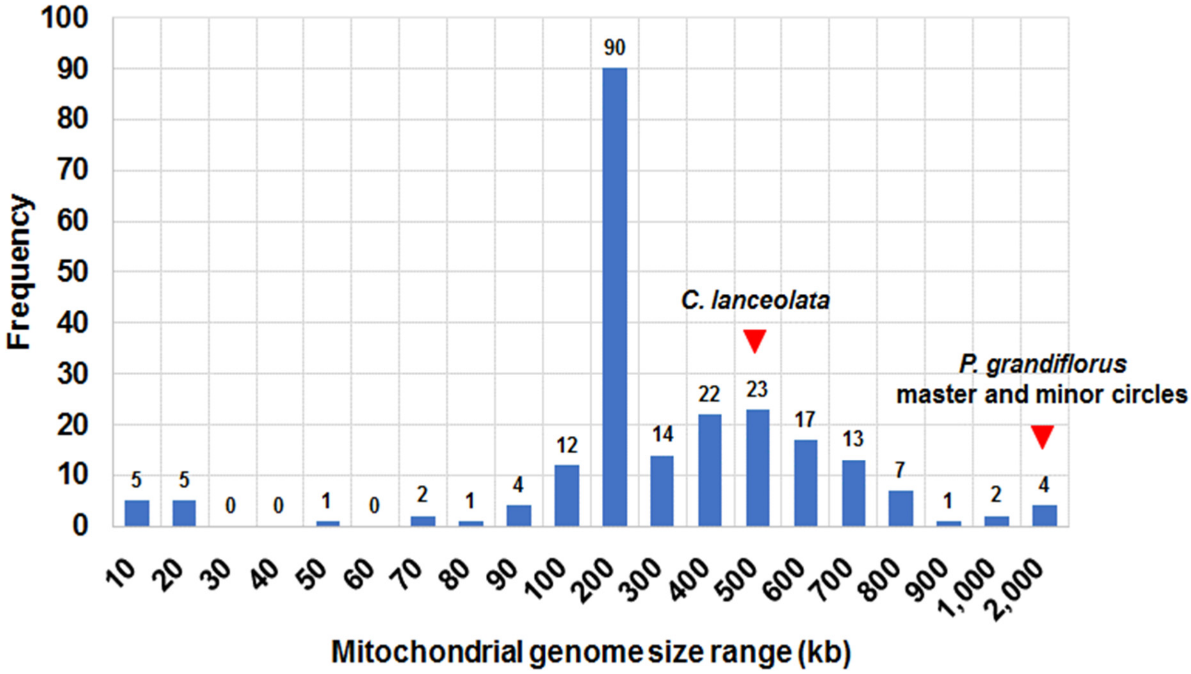

Supplementary Figure S5. Size distribution of mitochondrial genomes of land plant species, based on the NCBI Organelle Genome database (https://www.ncbi.nlm.nih.gov/genome/browse/?report=5#!/organelles/). The 223 mitochondrial genomes of land plant species have been deposited into the database.

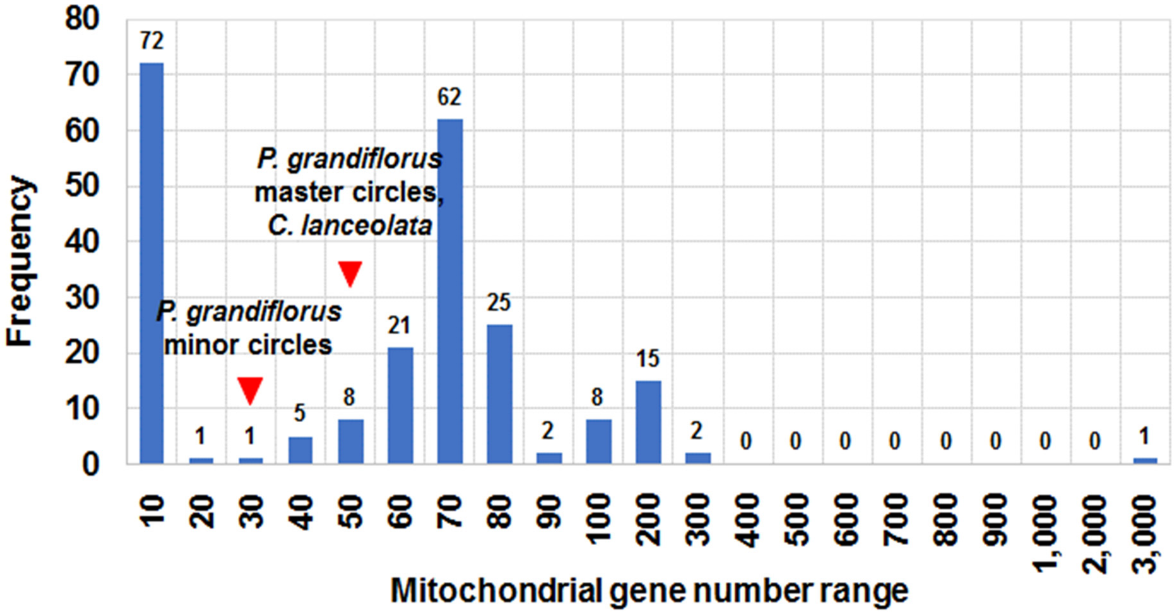

Supplementary Figure S6. Numbers of annotated genes in assembled mitochondrial genomes of land plants, as revealed by the NCBI Organelle Genome database.
