# Supplementary material for: Assembly of the Mitochondrial Genome in the Campanulaceae Family Using Illumina Low-Coverage Sequencing
Source: Genes (Basel). 2018 Jul 30;9(8):383. doi: 10.3390/genes9080383 (PMC6116063; doi:10.3390/genes9080383)
Supplement: Supplementary file 1 [file genes-09-00383-s001.zip › Supplementary-tables-final.pdf]

Supplementary Tables

Supplementary Table S1. Overview of raw and trimmed data.

Data type	*P. grandiflorus*	*C. lanceolata*
Total raw data (reads)	138,425,472	26,721,308
″ (bases)	20,902,246,272	8,043,113,708
Used raw data (reads)[1]	25,000,000	26,721,308
″ (bases)[1]	3,775,000,000	8,043,113,708
Trimmed data (reads)	23,971,262	23,424,520
″ (bases)	3,184,086,619	5,899,157,094
Mapped reads[2]	673,914	403,704
Average coverage	79.05	161.4

[1] Extracted from total raw data using manual method (Linux command line).

[2]Mitochondrial genome sequences were mapped by trimmed reads.

Supplementary Table S2. Comparison of four assemblers with two contig group.

**Category**		***P. grandiflorus***					***C. lanceolata***
Group	Type	Celera	SOAP[1]	SPAdes	CLC(master)[2]	CLC(minor)	CLC
Initial[3]	Number of contigs	132	58	355	172		143
	Total length (bp)	289,173	1,250,487	1,832,991	1,485,307		618,120
	Longest contig (bp)	32,924	158,662	99,337	134,690		85,287
	Shortest contig (bp)	1,010	103	142	201		207
	N50[5]	2,176	67,071	15,431	66,551		75,570
	L50[6]	38	7	21	8		4
Utilized[4]	Number of contigs	3	25	74	23	22	11
	Total length (bp)	37,027	1,192,214	1,223,031	1,193,802	1,064,991	430,454
	Longest contig (bp)	32,924	158,662	99,337	158,819	158,819	82,959
	Shortest contig (bp)	1,854	3,832	1,055	3,347	3,347	2,499
	N50	32,924	67,322	37,830	105,769	105,769	75,570
	L50	1	6	10	5	4	3

[1]SOAPdenovo assembler; [2]CLC novo assemble assembler; [3]Initial contigs of whole-genome; [4]Utilized contigs in the mitochondrial genome assembly; [5] Minimum contig length (bp) at 50% of the assembled genome; [6] Minimum number of contigs whose length sum equals N50.

Supplementary Table S3. Primers used for assembly validation.

Primer no.	Direction	Sequence (5'-3')	Size (bp)	Location	Locus
Validation_01	F	CGAATAGGCCTTCTCCCTTC	991	Intergenic	*trnF-GAA~mttB*
	R	AAAAAGAGCAAAAGCCAGCA			
Validation_02	F	GTTTGCCGGATTCAACAACT	969	Intergenic	*rpl10~trnM-CAU*
	R	GTGAGCGGGCAGAGTAAGTC			
Validation_03	F	TAGAATAGGGCGGCTTGCTA	913	Intergenic	*ccmFc~ccmB*
	R	TAGGCAGCCTGGTCTTGACT			
Validation_04	F	CCAGCCAAGAGGTTCTCAAG	995	Intergenic	*trnH-GUG~ccmC*
	R	GAATGAGTTCGCCTCTCAGG			
Validation_05	F	GGCGCCAGGAAATACAGATA	983	Intergenic	*trnQ-UUG~trnW-CCA*
	R	AGGAAAGGTTGTGCCCTTTT			
Validation_06	F	TCCTCCACTTCCTTCCCTCT	932	Intergenic	*trnM-CAU~rps13*
	R	AGGTCCCGGTCTTTGGTACT			
Validation_07	F	GGTTGCGAAGCAAGAAAGTC	1,000	Intergenic	*rps4~trnP-UGG*
	R	CGTTCCCAATACCTCGAAGA			
Validation_08	F	AATGCCTAGCTTCCGTCTCA	906	Intergenic	*atp6~ccmFn*
	R	TTCTTCGGATTCCATCTTGG			
Validation_09	F	ACTAGCCGTTGTGGTCATCC	925	Intergenic	*rrn5~rrn26*
	R	CGTGAGAAGGCCTAACTTCG			
Validation_10	F	CCCGCACTAGTGGAAGAGAG	975	Intergenic	*rps13~trnP-UGG*
	R	GAGCCTTGGCAGTTGTTCTC			
Validation_11	F	TGCCTAGCTAATTCGCACCT	900	Intergenic	*atp8~atp4*
	R	CCGGAGTTCTTGAGTGAAGC			
Validation_12	F	AGTTCGGCTTGGGTGATATG	966	Intergenic	*trnY-GUA~trnP-UGG*
	R	AGTAGGCCGAGATGCTTTCA			

Supplementary Table S4. Features of assembled mitochondrial genomes of *P. grandiflorus* (including master and minor circle) and *C. lanceolata* with *H. annuus*.

**Compositional Categories**	**P. master[1]**	**P. minor[2]**	***C. lanceolata***	***H. annuus*[3]**
Genome length (bp)	1,249,593	1,070,431	403,704	300,945
GC contents (%)	43.89	41.99	42.94	43.45
Total number of genes	56	42	54	42
Duplicated genes	10	6	7	0
Gene length sum (bp) [4]	40,368	31,359	40,030	29,488
Gene length including duplicated genes (bp)	42,337	31,840	40,544	29,488
Fraction of genome occupied by genes (%)	3.23	2.93	9.91	9.80
Fraction including duplicated genes (%)	3.39	2.97	10.04	9.80
Number of protein-coding genes	32	23	31	25
Total length of protein-coding sequences (bp)	27,168	18,306	28,042	22,560
Ratio of total protein-coding gene length (%)	2.17	1.17	6.94	7.49
Average exon length (bp)	603	572	596	867
Number of spliced protein-coding genes	3	3	3	0
Number of trans-spliced protein-coding genes	2	1	2	0
Number of non-spliced protein-coding genes	27	19	26	25
Number of transfer RNAs (tRNAs)	11	10	13	14
Number of ribosomal RNAs (rRNAs)	3	3	3	3

[1]Master circle of mitochondrial genome in the *P. grandiflorus.;* [2]Minor circle of mitochondrial genome in the *P. grandiflorus.;* [3]Features of the mitochondrial genome of reported *Helianthus annuus*.[19]; [4]Calculations include intron regions. For trans-spliced genes, only exons were included.

Supplementary Table S5. Repeat regions identified in the *P. grandiflorus* (including master and minor circle), *C. lanceolata* and *H. annuus*.

No.	Length	First region	Second region	Direction	Location	Locus
			P. grandiflorus (master)			
1	21,751	157411-179162	1227842-1249593	Forward	Intergenic	*rps4~trnP-UGG*
2	3,827	7548-11375	617189-621016	Forward	Intergenic	*trnF-GAA~mttB*
3	1,165	292904-294069	553231-554396	Forward	Intergenic	*atp6~ccmFn*
4	812	393692-394504	666638-667450	Forward	Intergenic	*nad2~ccmFc*
5	375	376633-377008	1146054-1146429	Reverse	Intergenic	*ccmFn~nad2*
6	294	1050510-1050804	1220732-1221026	Forward	Intergenic	*nad1~trnP-UGG*
7	283	310348-310631	1130016-1130299	Reverse	Intergenic	*atp6~ccmFn*
8	262	113559-113821	552980-553242	Forward	Intergenic	*cox1~nad1*
9	258	290486-290744	917521-917779	Forward	Intergenic	*atp6~ccmFn*
10	168	636372-636540	840709-840877	Forward	Intergenic	*rps7~trnC-GCA*
11	126	206419-206545	629276-629402	Forward	Intergenic	*trnM-CAU~atp6*
12	119	7787-7906	284375-284494	Reverse	Intergenic	*trnF-GAA~mttB*
13	119	284375-284494	617428-617547	Reverse	Intergenic	*atp6~ccmFn*
14	115	852968-853083	911571-911686	Reverse	Intergenic	*rrn5~rrn26*
15	113	29760-29873	629885-629998	Forward	Intergenic	*trnF-GAA~mttB*
16	111	433932-434043	1123837-1123948	Forward	Intergenic	*cob~atp9*
17	109	1149546-1149655	1200513-1200622	Forward	Intergenic	*atp8~atp4*
18	103	263397-263500	773470-773573	Forward	Intergenic	*atp6~ccmFn*
19	101	180385-180486	325390-325491	Reverse	Intergenic	*trnF-GAA~trnQ-UUG*
			P. grandiflorus (minor)			
1	1,165	113742-114907	374069-375234	Forward	Intergenic	*atp6~ccmFn*
2	812	214530-215342	487476-488288	Forward	Intergenic	*nad2~ccmFc*
3	375	197471-197846	966892-967267	Reverse	Intergenic	*ccmFn~nad2*
4	294	871348-871642	1041570-1041864	Forward	Intergenic	*rps13~trnP-UGG*
5	283	131186-131469	950854-951137	Reverse	Intergenic	*atp6~ccmFn*
6	258	111324-111582	738359-738617	Forward	Intergenic	*atp6~ccmFn*
7	168	457210-457378	661547-661715	Forward	Intergenic	*rps7~trnC-GCA*
8	126	27257-27383	450114-450240	Forward	Intergenic	*trnM-CAU~atp6*
9	119	105213-105332	438266-438385	Reverse	Intergenic	*atp6~ccmFn*
10	115	673806-673921	732409-732524	Reverse	Intergenic	*rrn5~rrn26*
11	111	254770-254881	944675-944786	Forward	Intergenic	*cob~atp9*
12	109	970384-970493	1021351-1021460	Forward	Intergenic	*atp8~atp4*
13	103	84235-84338	594308-594411	Forward	Intergenic	*atp6~ccmFn*
14	101	1223-1324	146228-146329	Reverse	Intergenic	*trnF-GAA~trnQ-UUG*

C. lanceolata						
1	2,317	155885-158202	239401-241718	Reverse	Intergenic	*cox1~trnM-CAU*
2	705	80094-80799	317242-317947	Reverse	Intergenic	*trnH-GUG~rrn18*
3	496	35230-35726	88389-88885	Reverse	Intergenic	*nad5~ccmC*
4	319	57769-58088	115663-115982	Forward	Genic	*cox2*
5	282	51166-51448	272464-272746	Forward	Intergenic	*trnF-GAA~cox2*
6	230	101223-101453	157996-158226	Forward	Intergenic	*rpl16~trnM-CAU*
7	206	101223-101429	239401-239607	Reverse	Intergenic	*rpl16~trnM-CAU*
8	170	51599-51769	352153-352323	Forward	Intergenic	*trnF-GAA~cox2*
9	131	116655-116786	290160-290291	Reverse	Intergenic	*nad6~nad1*
10	112	126572-126684	367713-367825	Reverse	Intergenic	*nad1~trnP-CGG*
11	102	35348-35450	355994-356096	Reverse	Intergenic	*nad5~ccmC*
12	102	88665-88767	355994-356096	Forward	Intergenic	*atp9~rps3*
H. annus						
1	5533	51681-57214	288011-293544	Forward	Intergenic	*trnD~trnK*
2	4347	57215-61562	293545-297892	Forward	Intergenic	*trnD~trnK*
3	2614	62000-64614	298331-300945	Forward	Intergenic	*trnK~ccmB*
4	722	36392-37114	189928-190650	Forward	Genic	*atp8*
5	450	172459-172909	245601-246051	Reverse	Intergenic	*trnS~cob*
6	439	61566-62005	297898-298337	Forward	Intergenic	*trnD~trnK*
7	272	122001-122273	223609-223881	Reverse	Genic	*rps4*
8	224	36539-36763	202672-202896	Reverse	Intergenic	*nad4L~atp8*
9	224	190075-190299	202672-202896	Reverse	Intergenic	*cob~ccmFc*
10	203	153070-153273	282999-283202	Forward	Intergenic	*rps13~nad6*
11	166	32211-32377	259635-259801	Reverse	Intergenic	*nad4L~atp8*
12	162	35511-35673	268977-269139	Forward	Intergenic	*nad4L~atp8*
13	153	149614-149767	284507-284660	Reverse	Intergenic	*rps13~nad6*
14	135	191176-191311	260792-260927	Forward	Intergenic	*cob~ccmFc*
15	124	35353-35477	268819-268943	Forward	Intergenic	*nad4L~atp8*
16	104	106127-106231	268924-269028	Forward	Genic	*coxI*

Supplementary Table S6. Tandem repeat sequences identified in the *P. grandiflorus* (including master and minor circle), *C. lanceolata* and *H. annuus*.

No.	Position	Consensus size (bp)	Copy number	Matches (%)	Location	Locus
		P. grandiflorus (master)				
1	8912-8952	18	2	100	Intergenic	*trnF-GAA ~ trnK-UUU*
2	136290-136325	18	2	94	Intergenic	*nad1 ~ nad6*
3	144290-144314	12	2	100	Intergenic	*rps4 ~ trnP-UGG*
4	238733-238763	15	2	93	Intergenic	*atp6 ~ ccmFn*
5	299800-299874	24	3	71	Intergenic	*atp6 ~ ccmFn*
6	352897-352942	23	2	91	Intergenic	*ccmFn ~ CDS*
7	454178-454223	20	2	79	Intergenic	*atp9 ~ CDS*
8	534282-534321	21	1	84	Intergenic	*nad7 ~ trnH-GUG*
9	544270-544295	13	2	100	Intergenic	*nad7 ~ trnH-GUG*
10	564556-564605	25	2	88	Intergenic	*nad7 ~ trnH-GUG*
11	564558-564611	25	2	82	Intergenic	*nad7 ~ trnH-GUG*
12	581801-581827	13	2	100	Intergenic	*trnH-GUG ~ ccmC*
13	618553-618593	18	2	100	Intergenic	*ccmC ~ rps7*
14	636631-636659	14	2	100	Intergenic	*rps7 ~ trnC-GCA*
15	645888-645915	14	2	100	Intergenic	*rps7 ~ trnC-GCA*
16	687274-687298	12	2	100	Intergenic	*nad2 ~ trnE-UUC*
17	763642-763680	18	2	86	Intergenic	*trnQ-UUG ~ trnW-CCA*
18	878356-878406	21	2	93	Intergenic	*rrn5 ~ rrn26*
19	910965-910990	13	2	100	Intergenic	*rrn5 ~ rrn26*
20	932098-932131	17	2	94	Intergenic	*rrn5 ~ rrn26*
21	962293-962329	18	2	94	Intergenic	*rrn5 ~ rrn26*
22	1019361-1019390	15	2	93	Intergenic	*trnM-CAU ~ rps13*
23	1046236-1046302	29	2	100	Intergenic	*rps13 ~ nad1*
24	1067143-1067177	17	2	88	Intergenic	*trnP-UGG ~ nad4*
25	1074865-1074890	13	2	100	Intergenic	*trnP-UGG ~ nad4*
26	1120590-1120615	13	2	100	Intergenic	*trnL-CAA ~ sdh4*
27	1141484-1141509	13	2	100	Intergenic	*atp8 ~ atp4*
		P. grandiflorus (minor)				
1	59571-59601	15	2	93	Intergenic	*atp6~ccmFn*
2	120638-120712	24	3	71	Intergenic	*atp6~ccmFn*
3	173735-173780	23	2	91	Intergenic	*ccmFn~nad2*
4	275016-275061	20	2	79	Intergenic	*atp9~nad7*
5	355120-355159	21	1	84	Intergenic	*nad7~trnH-GUG*
6	365108-365133	13	2	100	Intergenic	*nad7~trnH-GUG*
7	385394-385443	25	2	88	Intergenic	*nad7~trnH-GUG*
8	385396-385449	25	2	82	Intergenic	*nad7~trnH-GUG*
9	402639-402665	13	2	100	Intergenic	*trnH-GUG~ccmC*

10	439391-439431	18	2	100	Intergenic	*ccmC~rps7*
11	457469-457497	14	2	100	Intergenic	*rps7~trnC-GCA*
12	466726-466753	14	2	100	Intergenic	*rps7~trnC-GCA*
13	508112-508136	12	2	100	Intergenic	*nad2~trnE-UUC*
14	584480-584518	18	2	86	Intergenic	*trnQ-UUG~trnW-CCA*
15	699194-699244	21	2	93	Intergenic	*rrn5~rrn26*
16	731803-731828	13	2	100	Intergenic	*rrn5~rrn26*
17	752936-752969	17	2	94	Intergenic	*rrn5~rrn26*
18	783131-783167	18	2	94	Intergenic	*rrn5~rrn26*
19	840199-840228	15	2	93	Intergenic	*trnM-CAU~rps13*
20	867074-867140	29	2	100	Intergenic	*rps13~trnP-UGG*
21	887981-888015	18	2	88	Intergenic	*trnP-UGG~nad4*
22	895703-895728	13	2	100	Intergenic	*trnP-UGG~nad4*
23	941428-941453	13	2	100	Intergenic	*trnL-CAA~sdh4*
24	962322-962347	13	2	100	Intergenic	*atp8~atp4*
		C. lanceolata				
1	30-60	16	2	93	Intergenic	*nad5~trnE-UUC*
2	7875-7906	15	2	94	Intergenic	*trnE-UUC~nad5*
3	13157-13191	17	2	100	Intergenic	*trnE-UUC~nad5*
4	26164-26235	22	3	62	Intergenic	*trnE-UUC~nad5*
5	26149-26250	48	2	98	Intergenic	*trnE-UUC~nad5*
6	31775-31815	21	2	85	Intergenic	*nad5~ccmC*
7	37597-37711	48	2	91	Intergenic	*nad5~ccmC*
8	45773-45851	39	2	95	Intergenic	*trnF-GAA~cox2*
9	56730-56771	21	2	90	Intergenic	*trnF-GAA~cox2*
10	57028-57069	8	5	81	Intergenic	*trnF-GAA~cox2*
11	57028-57069	15	2	80	Intergenic	*trnF-GAA~cox2*
12	57029-57070	21	2	86	Intergenic	*trnF-GAA~cox2*
13	57505-57578	18	4	91	Intergenic	*trnF-GAA~cox2*
14	62758-62785	14	2	100	Intergenic	*cox2~cob*
15	65201-65340	26	5	75	Intergenic	*cob~nad1*
16	65198-65346	52	2	84	Intergenic	*cob~nad1*
17	65728-65756	14	2	100	Intergenic	*cob~nad1*
18	71527-71560	17	2	100	Intergenic	*cob~nad1*
19	85852-85888	14	2	87	Intergenic	*rrn5~atp9*
20	85926-86004	38	2	88	Intergenic	*rrn5~atp9*
21	149477-149509	15	2	88	Intergenic	*cox1~trnM-CAU*
22	157953-157982	14	2	100	Intergenic	*cox1~trnM-CAU*
23	175386-175417	16	2	100	Intergenic	*nad2~nad2*
24	180102-180126	13	1	100	Intergenic	*nad2~ccmFc*
25	184102-184130	13	2	100	Intergenic	*nad2~ccmFc*
26	185366-185424	26	2	79	Intergenic	*nad2~ccmFc*
27	235289-235330	18	2	91	Genic	*rrn26*

28	236608-236637	14	2	93	Intergenic	*rrn26~nad5*
29	239622-239651	14	2	100	Intergenic	*trnM-CAU~rps12*
30	285657-285694	19	2	94	Intergenic	*trnF-GAA~rps7*
31	297320-297366	23	2	95	Intergenic	*rps7~trnC-GCA*
32	341818-341842	13	1	100	Intergenic	*atp6~nad7*
		H. annus				
1	6193-6232	9	4	87	Intergenic	*trnY~trnN*
2	6187-6243	16	3	81	Intergenic	*trnY~trnN*
3	10993-11028	7	5	93	Intergenic	*trnC~ccmC*
4	10991-11028	12	3	88	Intergenic	*trnC~ccmC*
5	10981-11028	18	2	90	Intergenic	*trnC~ccmC*
6	23294-23318	12	2	100	Intergenic	*ccmC~atp4*
7	42396-42421	7	3	100	Intergenic	*coxIII~rpl5*
8	42397-42446	21	2	83	Intergenic	*coxIII~rpl5*
9	42418-42447	15	2	93	Intergenic	*coxIII~rpl5*
10	54941-54988	24	2	100	Intergenic	*trnD~trnK*
11	55786-55832	20	2	92	Intergenic	*trnD~trnK*
12	60607-60641	18	1	94	Intergenic	*trnD~trnK*
13	61641-61686	21	2	96	Intergenic	*trnD~trnK*
14	95812-95890	39	2	95	Intergenic	*trnE~coxI*
15	108220-108265	24	1	86	Intergenic	*coxI~nad5*
16	120658-120691	17	1	88	Intergenic	*atp9~rps4*
17	129277-129459	46	4	98	Genic	*rrn26*
18	129278-129460	91	2	100	Genic	*rrn26*
19	131171-131213	18	2	81	Genic	*rrn26*
20	132823-132866	21	2	95	Intergenic	*rrn26~rrn5*
21	159122-159155	17	2	100	Intergenic	*rps13~nad6*
22	160575-160638	25	2	100	Intergenic	*rps13~nad6*
23	168165-168204	20	2	100	Intergenic	*rps13~nad6*
24	186296-186375	31	2	75	Intergenic	*trnS~cob*
25	187081-187206	45	2	91	Intergenic	*trnS~cob*
26	189708-189754	21	2	96	Intergenic	*cob~ccmFc*
27	189720-189765	21	2	84	Intergenic	*cob~ccmFc*
28	198748-198787	16	2	92	Intergenic	*cob~ccmFc*
29	204820-204850	14	2	94	Intergenic	*atp1~ccmFn*
30	208565-208621	22	2	97	Intergenic	*atp1~ccmFn*
31	222470-222531	31	2	100	Intergenic	*ccmFn~ccmFn*
32	233450-233489	18	2	86	Intergenic	*rpl16~matR*
33	242359-242410	27	1	92	Intergenic	rpl16~matR
34	271932-271984	24	2	100	Intergenic	atp6~trnK
35	273281-273348	34	2	100	Intergenic	atp6~trnK
36	273611-273681	35	2	100	Intergenic	atp6~trnK
37	291271-291318	24	2	100	Intergenic	atp6~trnK

38	292116-292162	20	2	92	Intergenic	atp6~trnK
39	296937-296971	18	1	94	Intergenic	atp6~trnK
40	297973-298018	21	2	96	Intergenic	atp6~trnK
